# Supplementary material for: The Effects of 12-Week Prebiotic Supplementation on General Wellness and Exercise-Induced Gastrointestinal Symptoms in Recreationally Trained Endurance Athletes: A Triple-Blind Randomised Controlled Pilot Trial
Source: Nutrients. 2025 Oct 28;17(21):3390. doi: 10.3390/nu17213390 (PMC12610699; doi:10.3390/nu17213390)
Supplement: Supplementary file 1 [file nutrients-17-03390-s001.zip › nutrients-3933698-supplementary.pdf]

File S1: Detailed results write-up

The effects of group (A vs. B), time (pre vs. post), and their interaction on psychological, physiological, and lifestyle-related variables were examined using linear mixed models (LMM). Accordingly,

The LMM revealed a significant main effect of time on Cognitive_A_State, with a large decrease from pre to post (β = -17.56, $p < 0.001$, 95% CI [-20.37, -14.75]). However, there was no significant effect of group (β = -0.11, p = 0.949, 95% CI [-3.57, 3.34]), and no significant interaction between group and time (β = 0.11, p = 0.954, 95% CI [-3.66, 3.89]). The model showed a high conditional R^2 of 0.912, indicating that a substantial proportion of the variability in Cognitive_A_State was explained by the model. The marginal R^2 was 0.857, suggesting that the fixed effects alone accounted for a significant amount of the variance.

The LMM revealed no significant effects for Somatic_A_State. Specifically, the main effect of group was not significant (β = -0.67, p = 0.647, 95% CI [-3.56, 2.22]), there was no significant main effect of time (β = 0.44, p = 0.639, 95% CI [-1.47, 2.35]), and the interaction between group and time was not significant (β = -0.44, p = 0.739, 95% CI [-3.06, 2.17]). The model's conditional R^2 was 0.854, indicating that a substantial proportion of the variability in Somatic_A_State was explained by the model. The marginal R^2 was 0.780, suggesting that the fixed effects alone accounted for a significant portion of the variance.

The LMM revealed no significant effects for State_Self_Confidence. Specifically, the main effect of group (A vs. B) was not significant (β = -2.44, p = 0.230, 95% CI [-6.46, 1.57]), and there was no significant main effect of time (β = -0.22, p = 0.881, 95% CI [-3.11, 2.67]). Additionally, the interaction between group and time was not significant (β = -0.44, p = 0.832, 95% CI [-4.25, 3.36]). The model's conditional R^2 was 0.876, indicating that a substantial proportion of the variability in State_Self_Confidence was explained by the model. The marginal R^2 was 0.842, suggesting that the fixed effects alone accounted for a significant portion of the variance.

The linear mixed model (LMM) was not appropriate for Overall_Gut_Discomfort due to a singular fit and a lack of variability in the random effects (Variance = 0.00). As a result, a linear model (LM) was used for analysis. The LM showed that the main effect of group approached significance (β = -1.11, p = 0.073, 95% CI [-2.30, 0.07]), while the main effect of time (β = -1.00, p = 0.127, 95% CI [-2.32, 0.32]) and the interaction between group and time (β = 1.11, p = 0.227, 95% CI [-0.67, 2.88]) were not significant. The overall model was not significant ($F(3, 32) = 1.33$, p = 0.2808).

The LMM for Upper_GIS revealed that the intercept was significant (β = 4.78, p = 0.016, 95% CI [0.77, 8.80]). However, there were no significant effects for group (β = -3.44, p = 0.207, 95% CI [-8.92, 2.04]), time (β = -1.89, p = 0.456, 95% CI [-6.19, 2.41]), or the interaction between group and time (β = 1.78, p = 0.618, 95% CI [-5.47, 9.03]). The model's conditional R^2 was 0.199, suggesting that a small proportion of the variability in Upper_GIS was explained by the model, and the marginal R^2 was 0.062, indicating that the fixed effects alone accounted for a limited amount of the variance.

The LMM for Lower_GIS indicated that the intercept was significant (β = 6.22, $p = 0.005$, 95% CI [1.99, 10.45]). However, the effects of group (β = -3.22, $p = 0.285$, 95% CI [-9.24, 2.80]), time (β = -4.11, $p = 0.129$, 95% CI [-9.33, 1.10]), and the interaction between group and time (β = 1.67, $p = 0.654$, 95% CI [-5.90, 9.24]) were not significant. The model's conditional R^2 was 0.316, indicating that a moderate proportion of the variability in Lower_GIS was explained by the model, and the marginal R^2 was 0.100, suggesting that the fixed effects alone accounted for a smaller portion of the variance.

The LMM for Other_GIS revealed that the intercept was marginally significant (β = 1.89, $p = 0.063$, 95% CI [-0.01, 3.79]). However, there were no significant effects for group (β = -0.56, $p = 0.691$, 95% CI [-3.46, 2.34]), time (β = -0.33, $p = 0.774$, 95% CI [-2.50, 1.84]), or the interaction between group and time (β = 0.00, $p = 1.000$, 95% CI [-3.23, 3.23]). The model's conditional R^2 was 0.327, indicating that a moderate proportion of the variability in Other_GIS was explained by the model, while the marginal R^2 was 0.012, suggesting that the fixed effects alone accounted for a very small portion of the variance.

The LMM for q3_SLEEP revealed a significant intercept (β = 17.39, $p < 0.001$, 95% CI [11.00, 23.78]). However, the effects of group (β = -3.57, $p = 0.400$, 95% CI [-12.86, 5.72]), time (β = -5.02, $p = 0.072$, 95% CI [-10.45, 0.42]), and the interaction between group and time (β = 0.04, $p = 0.992$, 95% CI [-7.00, 7.08]) were not significant. The model's conditional R^2 was 0.683, indicating that a substantial proportion of the variability in q3_SLEEP was explained by the model, while the marginal R^2 was 0.131, suggesting that the fixed effects alone accounted for a smaller portion of the variance.

The LMM for q4_SLEEP revealed a significant intercept (β = 2.14, $p = 0.005$, 95% CI [0.72, 3.57]), but the effects of group (β = -1.07, $p = 0.257$, 95% CI [-2.97, 0.84]), time (β = -0.80, $p = 0.093$, 95% CI [-1.72, 0.11]), and the interaction between group and time (β = 1.17, $p = 0.072$, 95% CI [-0.05, 2.39]) were not fully significant. The model had a conditional R^2 of 0.792, indicating that the model explained a significant portion of the variability in q4_SLEEP. However, the marginal R^2 was lower at 0.048, suggesting that the fixed effects alone accounted for a smaller proportion of the variance. This highlights the importance of including random effects in the analysis to capture individual variability.

The LMM for q8_SLEEP revealed a significant intercept (β = 427.06, $p < 0.001$, 95% CI [360.89, 493.23]), but the effects of group (β = 43.65, $p = 0.346$, 95% CI [-48.45, 135.76]), time (β = -55.65, $p = 0.101$, 95% CI [-121.79, 10.48]), and the interaction between group and time (β = 44.90, $p = 0.320$, 95% CI [-46.34, 136.13]) were not statistically significant. The model had a conditional R^2 of 0.614, indicating that a moderate proportion of the variability in q8_SLEEP was explained by the model. The marginal R^2 was 0.164, suggesting that the fixed effects alone accounted for a significant portion of the variance, but other factors such as random effects likely contributed as well.

The LMM for q9_SLEEP revealed a significant intercept (β = 3.27, $p < 0.001$, 95% CI [2.67, 3.87]), but the effects of group (β = 0.54, $p = 0.192$, 95% CI [-0.28, 1.36]), time (β = -0.47, $p = 0.192$, 95% CI [-1.17, 0.23]), and the interaction between group and time (β = 0.52, $p = 0.285$, 95% CI [-0.49, 1.54]) were not statistically significant. The model had a conditional R^2 of 0.477, indicating that a moderate proportion of the variability in q9_SLEEP was explained by the model. The marginal R^2 was 0.241, suggesting that the fixed effects alone accounted for a notable portion of the variance.

The LMM for q10_SLEEP revealed a significant intercept (β = 2.88, $p < 0.001$, 95% CI [2.21, 3.55]), but the effects of group (β = 0.59, p = 0.198, 95% CI [-0.30, 1.48]), time (β = -0.20, p = 0.470, 95% CI [-0.79, 0.39]), and the interaction between group and time (β = 0.11, p = 0.768, 95% CI [-0.65, 0.88]) were not statistically significant. The model had a conditional R^2 of 0.694, indicating that a substantial portion of the variability in q10_SLEEP was explained by the model, while the marginal R^2 was 0.134, suggesting that the fixed effects alone accounted for a moderate portion of the variance.

The LMM for q12a_SLEEP revealed a significant intercept (β = 0.60, p = 0.009, 95% CI [0.13, 1.08]), but the effects of group (β = -0.14, p = 0.635, 95% CI [-0.72, 0.44]) and time (β = -0.38, p = 0.068, 95% CI [-0.77, 0.01]) were not fully significant. However, the interaction between group and time approached significance (β = 0.52, p = 0.065, 95% CI [-0.02, 1.06]). The model had a conditional R^2 of 0.613, indicating that a substantial portion of the variability in q12a_SLEEP was explained by the model. The marginal R^2 was 0.072, suggesting that the fixed effects alone accounted for a smaller proportion of the variance.

The LMM for q13a_SLEEP revealed a significant intercept (β = 2.79, p = 0.003, 95% CI [1.06, 4.51]), but the effects of group (β = 0.09, p = 0.938, 95% CI [-2.20, 2.39]), time (β = -0.12, p = 0.835, 95% CI [-1.31, 1.07]), and the interaction between group and time (β = 1.26, p = 0.125, 95% CI [-0.24, 2.74]) were not significant. The model had a conditional R^2 of 0.774, indicating that a substantial portion of the variability in q13a_SLEEP was explained by the model. The marginal R^2 was 0.063, suggesting that the fixed effects alone accounted for a small portion of the variance.

The LMM for q1_IPAQ revealed a significant intercept (β = 3.17, p = 0.003, 95% CI [1.33, 5.00]), but the effects of group (β = -0.33, p = 0.789, 95% CI [-2.85, 2.20]), time (β = -0.17, p = 0.715, 95% CI [-1.10, 0.77]), and the interaction between group and time (β = 0.67, p = 0.312, 95% CI [-0.64, 1.98]) were not significant. The model had a conditional R^2 of 0.867, indicating that a large proportion of the variability in q1_IPAQ was explained by the model. However, the marginal R^2 was 0.008, suggesting that the fixed effects alone accounted for a very small portion of the variance, highlighting the importance of the random effects in explaining the variability.

The LMM for q2_IPAQ revealed a significant intercept (β = 40.00, p = 0.020, 95% CI [7.99, 72.01]), but the effects of group (β = -7.17, p = 0.751, 95% CI [-59.77, 45.42]), time (β = 2.17, p = 0.897, 95% CI [-30.42, 34.76]), and the interaction between group and time (β = 34.50, p = 0.163, 95% CI [-16.42, 85.43]) were not significant. The model had a conditional R^2 of 0.522, indicating that the model explained a moderate portion of the variability in q2_IPAQ. The marginal R^2 was 0.120, suggesting that the fixed effects alone accounted for a smaller portion of the variance.

The LMM for q3_IPAQ revealed a marginally significant intercept (β = 1.50, p = 0.055, 95% CI [-0.01, 3.01]), but the effects of group (β = 0.50, p = 0.638, 95% CI [-1.61, 2.61]), time (β = 0.33, p = 0.738, 95% CI [-1.77, 2.43]), and the interaction between group and time (β = -0.50, p = 0.723, 95% CI [-3.48, 2.48]) were not significant. The model had a conditional R^2 of 0.148, indicating that a small portion of the variability in q3_IPAQ was explained by the model. The marginal R^2 was 0.010, suggesting that the fixed effects alone accounted for a very small portion of the variance.

The linear mixed model (LMM) was not appropriate for q4_IPAQ due to a singular fit and the lack of variability in the random effects (Variance = 0.00). Therefore, a linear model (LM) was used for analysis. The LM showed non-significant results for the intercept (β = 15.33, p = 0.229, 95% CI [-10.15, 40.81]), group (β = 18.00, p = 0.316, 95% CI [-18.58, 54.58]), time (β = 23.83, p = 0.188, 95% CI [-12.56, 60.23]), and the interaction between group and time (β = -22.17, p = 0.381, 95% CI [-

71.99, 27.65]). The model had a marginal R^2 of 0.086, but due to the singular fit, the conditional R^2 could not be calculated.

The linear mixed model (LMM) was not appropriate for q5_IPAQ due to a singular fit and the lack of variability in the random effects (Variance = 0.00). Therefore, a linear model (LM) was used for analysis. The LM yielded non-significant results for the intercept (β = 5.17, p < 0.001, 95% CI [3.69, 6.64]), group (β = 1.00, p = 0.344, 95% CI [-1.09, 3.09]), time (β = -4.71e-15, p = 1.000, 95% CI [-2.05, 2.05]), and the interaction between group and time (β = -1.33, p = 0.372, 95% CI [-4.36, 1.69]). The model had a marginal R^2 of 0.075, but the conditional R^2 could not be calculated due to the singular fit.

The linear mixed model (LMM) for q6_IPAQ showed non-significant results for the intercept (β = 90.00, p = 0.295, 95% CI [-81.46, 261.46]), group (β = 20.00, p = 0.867, 95% CI [-234.25, 274.25]), time (β = -45.00, p = 0.565, 95% CI [-211.75, 121.75]), and the interaction between group and time (β = 168.33, p = 0.144, 95% CI [-57.09, 393.75]). The model had a conditional R^2 of 0.629, indicating that a substantial portion of the variability in q6_IPAQ was explained by the model, and a marginal R^2 of 0.109, suggesting that the fixed effects alone accounted for a smaller proportion of the variance.

The linear mixed model (LMM) for q7_IPAQ showed a significant intercept (β = 280.00, p < 0.001, 95% CI [170.91, 389.09]), but non-significant results for group (β = 75.00, p = 0.322, 95% CI [-107.56, 257.56]), time (β = 40.00, p = 0.392, 95% CI [-56.78, 136.78]), and the interaction between group and time (β = -60.00, p = 0.365, 95% CI [-195.64, 75.64]). The model had a conditional R^2 of 0.641, indicating that a substantial proportion of the variability in q7_IPAQ was explained by the model, and a marginal R^2 of 0.046, suggesting that the fixed effects alone accounted for a relatively small proportion of the variance.

The linear mixed model (LMM) for WEMWBS Total Score showed a significant intercept (β = 51.00, p < 0.001, 95% CI [46.01, 55.99]), but no significant effects for group (β = 0.25, p = 0.941, 95% CI [-6.78, 7.28]), time (β = 2.17, p = 0.242, 95% CI [-1.67, 6.00]), or the interaction between group and time (β = -0.42, p = 0.862, 95% CI [-5.47, 4.63]). The model had a conditional R^2 of 0.756, suggesting that a substantial proportion of the variability in WEMWBS Total Score was explained by the model, and a marginal R^2 of 0.025, indicating that the fixed effects alone accounted for a small proportion of the variance.

The linear mixed model (LMM) for the general stress score showed a significant intercept (β = 5.15, p < 0.001, 95% CI [3.76, 6.53]), but no significant effects for group (β = 0.67, p = 0.447, 95% CI [-1.14, 2.49]), time (β = 0.14, p = 0.796, 95% CI [-0.99, 1.27]), or the interaction between group and time (β = -0.60, p = 0.406, 95% CI [-2.14, 0.94]). The model had a conditional R^2 of 0.680, indicating that the model explained a substantial proportion of the variability in the general stress score, and a marginal R^2 of 0.026, suggesting that the fixed effects alone accounted for a small proportion of the variance.

The linear mixed model (LMM) for the recovery-related score revealed a significant intercept (β = 13.30, p < 0.001, 95% CI [10.52, 16.08]), but no significant effects for group (β = 1.10, p = 0.530, 95% CI [-2.48, 4.68]), time (β = 1.20, p = 0.278, 95% CI [-1.13, 3.53]), or the interaction between group and time (β = -1.58, p = 0.282, 95% CI [-4.64, 1.49]). The model had a conditional R^2 of 0.672, indicating that the model explained a substantial portion of the variability in the recovery-related score, and a marginal R^2 of 0.020, suggesting that the fixed effects alone accounted for a small proportion of the variance.

The linear mixed model (LMM) was not appropriate for the sport-specific score, as indicated by a singular fit and the lack of variability in the random effects (Variance = 0.00). Therefore, a linear model (LM) was used. The LM showed a significant main effect of group, with Group B scoring higher than Group A (β = 1.99, p = 0.026, 95% CI [0.25, 3.72]). However, there were no significant effects of time (β = 0.61, p = 0.505, 95% CI [-1.25, 2.47]) or the interaction between group and time (β = -0.07, p = 0.954, 95% CI [-2.53, 2.39]). The model had a marginal R^2 of 0.300, indicating a moderate proportion of variance explained by the fixed effects, but the conditional R^2 could not be computed due to a singular fit.

The linear mixed model (LMM) for the sport-specific recovery score did not reveal any significant effects. The main effect of group was not significant (β = 0.58, p = 0.751, 95% CI [-3.26, 4.42]), nor was the main effect of time (β = 1.29, p = 0.262, 95% CI [-1.11, 3.70]). The interaction between group and time was also not significant (β = -1.54, p = 0.309, 95% CI [-4.72, 1.64]). The marginal R^2 was 0.018, indicating that the fixed effects explained little variance, while the conditional R^2 was 0.682, suggesting that most of the variance was accounted for by random effects.
